# Reply to A Wittekind

**DOI:** 10.1093/jn/nxz336

**Published:** 2020-03-05

**Authors:** Esther Winters van Eekelen, Joline W J Beulens, Frits R Rosendaal, Renée de Mutsert

**Affiliations:** 1 From the Department of Clinical Epidemiology Leiden University Medical Center, Leiden, Netherlands; 2 Department of Epidemiology and Biostatistics, Amsterdam Public Health Research Institute, VU Medical Center, Amsterdam, Netherlands; 3 Julius Center for Health Sciences and Primary Care, University Medical Center Utrecht, Utrecht, Netherlands

Dear Editor:

We thank Wittekind for her interest in our recent work on the association between the consumption of alcoholic and nonalcoholic beverages and hepatic triglyceride content, and her comments regarding the challenges involved in interpreting dietary substitution models.

Wittekind doubts the presence of the associations of subtypes of the nonalcoholic beverages tea, milk, and sugar-sweetened beverages (SSBs) with liver fat content because they were nonsignificant. The standard errors of the estimates of the subtypes of beverages were indeed larger than those of the total alcoholic beverages and nonalcoholic beverages, in part because fewer people consume each subtype, but also due to fewer degrees of freedom in the multivariate models. Although the confidence intervals just included the null value (1.00), we based our interpretation on the strength and direction of associations instead of relying on statistical significance, as this is an arbitrary cutoff and the *P* value is a function of the sample size (1–2). Absence of statistical significance does not necessarily imply absence of association, and relying solely on statistical significance has been heavily criticized. Confidence intervals should therefore not be used as a proxy for the *P* value but rather as an estimate of the size and direction of the association. Because the directions of the associations are supported by previous research, including randomized clinical trials (3–5), we are confident to conclude that consumption of SSBs is associated with increased liver fat.

Although the results of the models not considering substitution answer the question, How is 1 extra serving of a certain beverage, on top of all consumed beverages, associated with liver fat, the substitution models define a contrast and answer the question, What happens with the liver fat content if a specific beverage is replaced by another? The results of these models cannot be directly compared. To answer our question as to what beverage someone who is advised to refrain from alcohol should replace it with, we also performed substitution analysis.

In summary, the main results of our study are asfollows:

1. Each additional serving/day of alcoholic beverages is associated with more liver fat (1.09 times; 95% CI: 1.05, 1.12).
2. Each additional serving/day of nonalcoholic beverages is associated with less liver fat (0.97 times; 95% CI: 0.95, 0.99).
3. Replacement of 1 serving of alcohol with 1 serving of nonalcoholic beverage was associated with less liver fat (0.90 times; 95% CI: 0.86, 0.94).
4. Isocaloric replacement of 5% of energy (En%) derived from alcoholic beverages with 5 En% derived from milk was associated with less liver fat (0.89 times; 95% CI: 0.81, 0.98). In contrast to these inverse associations, the association of the replacement of 5 En% of an alcoholic beverage with 5 En% of SSBs was 1.00 (95% CI: 0.91, 1.09).

We acknowledge that the results from these cross-sectional analyses are based on a modeled, and not an actual, exchange of alcoholic beverages with nonalcoholic beverages. Nevertheless, an effect estimate of 1.00 (95% CI: 0.91, 1.09) indicates that replacing 5 En% of alcoholic beverages with 5 En% of SSBs is associated with liver fat to the same extent as consuming 5 En% of alcoholic beverages, as that is the reference in this model. In other words, these results suggest that replacing alcohol with SSBs is equally associated with liver fat (and not with more, or with less, liver fat). We therefore agree with Wittekind that our results suggest that there may not be any benefit in replacing alcoholic beverages with SSBs in reducing liver fat, and also believe this lends further support to our findings that consumption of SSBs is associated with increased liver fat.

We regret being unclear in describing the interpretation of our results and are grateful for the opportunity to further elaborate on them.

## Notes

The Netherlands Epidemiology of Obesity study is supported by the participating Departments, the Division and the Board of Directors of the Leiden University Medical Center, and by the Leiden University, Research Profile Area “Vascular and Regenerative Medicine.” We acknowledge the support from the Netherlands Cardiovascular Research Initiative, an initiative with support of the Dutch Heart Foundation (CVON2014-02 ENERGISE), and from the Nutricia Research Foundation. This research was conducted within the Health Holland framework and financially supported by Unilever Research and Development Vlaardingen and Health-Holland.

Author disclosures: The authors report no conflicts of interest.
